# A Subtracted‐Added‐Divided Inversion Recovery (dSIR) Approach to Visualise the Effects of Microstructure on T1 Contrast in Human White Matter

**DOI:** 10.1002/nbm.70070

**Published:** 2025-05-29

**Authors:** Risto A. Kauppinen, Jeromy Thotland, Pramod K. Pisharady, Christophe Lenglet, Michael Garwood

**Affiliations:** ^1^ Department of Electric, Electronic and Mechanical Engineering University of Bristol Bristol UK; ^2^ Center for Magnetic Resonance Research University of Minnesota Minneapolis Minnesota USA

**Keywords:** added and divided (dSIR), microstructure, subtracted, T1 relaxation, white matter

## Abstract

Recent evidence has demonstrated that several white matter (WM) microstructural features, such as axon diameter, fibre configurations and fibre orientation in respect to the magnetic field influence T1 relaxation. The effects from microstructural features on T1 are small in size, thus, visualising the effects of WM microstructure remains challenging in standard T1 weighted MRI in vivo. Here, we have studied an algebraic approach involving subtraction, addition and division of closely spaced inversion time images in WM imaging, the so‐called dSIR approach. Images collected with short TI (300 ms at 3T and 600 ms at 7T) and long TI (600 ms at 3T and 1000 ms at 7T) with MP2RAGE MRI were combined using the dSIR processing. dSIR signal intensities were compared with absolute T1 images. We found that dSIR was linearly related with T1 relaxation time over approximately 200 ms both at 3T and 7T. The slope of the dSIR versus T1 plot was 1.6 times greater at 7T than at 3T indicative of higher dSIR contrast at 7T. dSIR contrast revealed WM tracts that are oriented with high angle (fibre‐to‐field angle > 75°), in addition, dSIR signal showed angular patterns that closely resembled those of T1 at both fields. The dSIR contrast due to intratissue T1 difference of order of ~50 ms generated by microstructural features, including axon fibre orientation as well as by the presence of large and giant axons in somato‐motor subsection of corpus callosum were visualised. It is concluded that dSIR signal mimics T1 and that the dSIR contrast is higher at 7T than at 3T; thus, the approach will help to visualise the effects of microstructure on T1 to evaluate WM integrity.

## Graphical abstract

TI images collected with short (300ms/600ms, 3T/7T) and long TI (600ms/1000ms, 3T/7T) were processed using the dSIR approach to visualise effects of white matter (WM) microstructure on T1 contrast. Short T1 resulting from relaxation anisotropy in WM tracts with close to perpendicular orientation to B0 was visualised by excellent dSIR contrast against adjacent WM [Figure A (3T) and B (7T)]. dSIR provides a means to visualise the effects of microstructure on T1 to evaluating WM integrity.

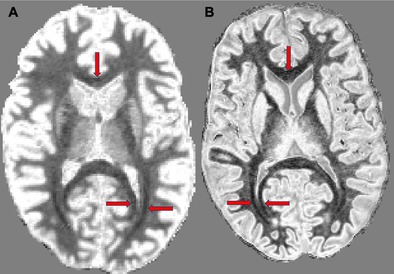

**Abbreviations**
*θ*_FB_
: fibre‐to‐field angle
AIR: added inversion recovery filter
BF: bipolar filter
CC: corpus callosum
CNR: contrast‐to‐noise ratio
DTI: diffusion tensor imaging
dSIR: subtracted‐added‐divided inversion recovery
GM: grey matter
HCP: Human Connectome Project
MASTIR: multiplied, added, subtracted, and fitted inversion recovery
mD: middle domain
MSE: mean squared error
MT: magnetisation transfer
PSNR: peak signal‐to‐noise‐ratio
SAD: subtracted, added, divided
SIR: subtracted inversion recovery filter
THB: Transient Hydrogen Bond
TI: difference of inversion times
TP: tissue property
WM: white matter

## 1 Introduction

The 3D MPRAGE T1‐weighted images [1] are perhaps the most commonly acquired brain scans owing to their good anatomical details and contrast between grey matter (GM) and white matter (WM). MPRAGE images are the backbone of morphometric analyses of GM and measurements of regional volumes, cortical thicknesses and shapes for both neuroscientific and clinical purposes. Two images acquired with a MP2RAGE method with widely different TIs enables improved T1 contrast in the brain by combining the two images. The two TI images by MP2RAGE are also used to compute absolute T1 images [2]. T1 MR signal in WM collected either by MPRAGE or MP2RAGE is chiefly influenced by macromolecules‐to‐water ratio and myelination with minor influence by biological iron. Magnetisation interaction, involving either chemical exchange or through space dipole interaction, between myelin and bulk water protons is the key physical element underpinning T1 relaxation in WM, the so‐called magnetisation transfer (MT) [3].

A growing body of evidence shows that WM microstructure beyond classical MT [4] modulates T1 relaxation [5, 6, 7, 8, 9]. R1 relaxation rate (= 1/T1) in WM is inversely proportional to axon diameter [5]. Similarly, axon fibre configuration and degree of structural anisotropy influence T1 (and hence, R1) [9, 10]. The effects of axon fibre orientation with respect to the magnetic field [6, 8, 9, 11, 12] have been demonstrated both at 3T and 7T. The angular patterns of T1 as measured either by variable flip angle [7] or MP2RAGE methods [12], show longer T1 in fibres running parallel to the field than in those perpendicular to B0. In addition to this, the T1 angular plots in images acquired by MP2RAGE show a broad long T1 feature centred at 40° both at 3T and 7T [12, 13]. The T1 angular patterns in vivo have been shown to be similar to those measured in ex vivo WM preparations [8, 13] following rotations around the B0 static field. The ex vivo data directly point to the relaxation anisotropy as the NMR physical underpinning of T1 angular dependency in WM [8, 13]. Restricted lateral diffusion of macromolecule‐bound protons in the long lipid molecules, such as in those in membranes and myelinated axons, modulates dipole–dipole interactions in neighbouring lipid molecules resulting in the orientation dependency of the longitudinal relaxation [14]. According to the so‐called transient hydrogen bond (THB) model transfer of the orientation dependent longitudinal relaxation of immobilised protons to MRI detectable water involves exchange of magnetisation through hydrogen‐bond‐driven structural order of dipole–dipole connections between immobile and mobile hydrogens [15]. The T1 angular patterns observed in WM [8, 13] are quantitatively explained by the descendant of the THB model, the so‐called Basic Transient Hydrogen model [16].

However, the effects of all the above‐mentioned WM microstructural factors on T1 are small, typically less than 5% of total T1, and hence difficult to visualise by standard T1 MR images. Recently, approaches were introduced to ‘amplify’ contrasts in inversion recover (IR) images based on algebraic processing of a pair of IR images [17, 18, 19]. The approaches are primarily targeted to enhance contrast in the tissue boundaries (partial voluming at a voxel scale), such as the GM/WM and GM/CSF interfaces, as well as to detect small changes in relaxation in brain lesions, that is, abnormalities in brain parenchyma associated with acute traumatic brain injury [20] and leucoencephalopathy [18]. An interesting application of an ultra‐short TE MRI sequence with IR nulling of water signal at 3T includes myelin imaging in MS patients suggesting that such an approach may be used to image WM microstructural features [21]. The protocols are designed to use the so‐called tissue property (TP) filters for algebraic processing of a set of TI images, that is, using subtracted, added, divided or multiplied image pairs, hence acronyms such as dSIR and MASTIR [18]. Amplification of IR contrast by up to 10‐fold has been achieved [18]. Here, we have used a subtracted‐added‐divided IR (dSIR) approach on a set of MP2RAGE scans at 3T and 7T. We generated images of the WM to examine the potential of dSIR to reveal small variations in T1 due to microstructural factors in healthy tissue, such as axon fibre orientation.

## 2 Methods

### 2.1 Human Subjects

The study protocol received ethical approval from the University of Minnesota Institutional Review Board. Six healthy volunteers (mean age 27 years, two females) consented to participate in the study. All six were scanned at both at 3T and 7T, 6–9 months apart.

### 2.2 MRI

A Siemens MAGNETOM Prisma 3T system with a 32‐channel head coil and a Siemens MAGNETOM 7T AS scanner with a Nova Medical 1 transmit/32 receive head coil were used. At 3T, diffusion MRI (dMRI) were acquired using the Human Connectome Project (HCP) Lifespan Protocol [22] with the parameters given in Table 1. All dMRI scans were acquired without angulation at scanner coordinates. A B0 field map was acquired using a spin echo EPI sequence with TR = 8000 ms, TE = 66 ms, 2 mm^3^ isotropic resolution. A B1 map was also acquired at resolution of 4 × 4 × 8 mm^3^ using the manufacturer's routine. At 7T, diffusion MRI were acquired using the HCP Young Adult Protocol [23] with the parameters shown in Table 1.

**TABLE 1:** Diffusion and MP2RAGE MRI acquisition parameters at 3T and 7T.

Parameter	3T dMRI	7T dMRI	3T MP2RAGE	7T MP2RAGE
Voxel size (mm)	1.5 × 1.5 × 1.5	1.05 × 1.05 × 1.05	1.25 × 1.25 × 1.25	0.9 × 0.9 × 0.9
Slices	92	128	3D	3D
TR (ms)	3230	7000	1850	3540
TE (ms)	89.2	71.2	1.69	1.49
TR (ms) in readout	—	—	3.6	3.2
Readout pulse	180°	180°	4° hard pulse	4° hard pulse
GRAPPA	—	3	3	3
Phase PF	—	—	6/8	6/8
Slice PF	—	—	6/8	6/8
Phase encoding	A ≫ P, P ≫ A	A ≫ P, P ≫ A	A ≫ P	A ≫ P
Gradient directions	197(AP), 197(PA)	143(AP), 143(PA)	—	—
*b* values (s/mm^2^)	1500, 3000	1000, 2000	—	—
*b* = 0 s/mm^2^ volumes	13(AP),17(PA)	11(AP),13(PA)	—	—
TI (ms) pairs acquired	—	—	200/1200, 300/900, 600/1500	300/1500, 600/2000, 1000/3000
Acquisition time (min:s)	22:38	39:20	three blocks of 5:10 each, total time 15:30	three blocks of 9:33 each, total time 28:06

*Note:* ‘dMRI’ stands for diffusion MRI.

An MP2RAGE sequence was used to acquire images for T1 mapping both at 3T and 7T, with the acquisition parameters given in Table 1. MP2RAGE images for T1 maps at 3T were acquired at isotropic voxel size of 1.25 mm^3^, while at 7T MP2RAGE images were acquired at isotropic voxels sizes of 0.9 mm^3^. Anatomical T1‐weighted MPRAGE images at both fields (acquired at 0.8 and 1.0 mm^3^ isotropic resolutions at 3T and 7T, respectively) were used to segment GM, WM and CSF spaces.

### 2.3 Image Processing

dMRI scans were corrected for distortions due to eddy currents, susceptibility‐induced off‐resonance artefacts and subject motion using TOPUP and EDDY in FSL [24, 25]. A DTI model was subsequently fitted to the corrected data using DTIFIT in FSL [26], to compute the DTI indices (FA, MD, V_1_, V_2_, V_3_) using *b* = 0 s/mm^2^ and *b* = 1500 s/mm^2^ images at 3T and *b* = 0 and *b* = 1000 s/mm^2^ at 7T. The general consensus is that the optimal b‐value lies within 700 and 1500 s/mm^2^, with 1000 s/mm^2^ being the most commonly used value [27]. Fibre‐to‐field angle maps (*θ*_FB_) maps were computed from the principal direction of diffusion using the principal eigenvector V_1_ images and direction of B0 as previously described [6].

T1 maps were computed using a mono‐exponential fitting technique as previously described [6]. Diffusion data were aligned to the T1 images by registering FA maps first to the R1 (R1 = 1/T1) images for 3T and 7T data using FLIRT in FSL [28]. The 2D plots of T1 and dSIR image intensity as a function of $\theta_{\mathrm{FB}}$ were computed in Matlab as previously described [6]. Both peak signal‐to‐noise‐ratio (PSNR) and contrast‐to‐noise‐ratio (CNR) were determined in the parieto‐occipital brain by measuring signal intensities in ROIs of 3 × 3 × 3 voxels in both tissue types. PSNR was computed using the formula as follows:

$$\text{PSNR}=10*\log_{10}\;\left(\frac{S_{\max}^{2}}{\mathrm{MSE}}\right)$$

Where $S_{\max}^{2}$ is maximal signal to power of 2 and MSE is the mean squared error [29]. CNR was calculated to dividing the signal intensity difference between the two tissue types by the square root of summed SDs to power 2.

### 2.4 dSIR Processing

MP2RAGE acquisition parameters (Table 1) were primarily designed to acquire the sets of TI image for computations of absolute T1 images. For dSIR processing a pair of TI images are used [17, 19, 20]. dSIR signal intensity is determined by TI image timings and T1 of the system [17], thus the TI images must be selected to provide best ‘dSIR contrast’ in the target T1 range. Typically, the window of TI times, the so‐called middle domain (= mD), will assure that [a] null points of target T1s fall favourable relative to the mD to minimise noise bias, and [b] the mD will be narrow to map the target T1s to the dSIR signal range of −1 to +1 as well as to provide a filter for separation of small T1 differences [30]. Target T1 values of this study, as measured by the MP2RAGE MRI, range from 770 to 860 ms at 3T and from 890 to 970 ms at 7T [9]. The effects of microstructure on T1 become measurable in WM with FA > 0.4, where T1 proceeds within the abovementioned ranges [9, 11, 12]. We therefore used TI = 300 ms and TI = 600 ms (difference in inversion times = ΔTI = 300 ms) at 3T and TI = 600 ms and TI = 1000 ms (ΔTI = 400 ms) in magnitude mode for dSIR processing. In TI 300/600 ms images WM is dark and GM bright while the opposite appearance of the two tissue types is evident in TI = 600/1000 ms images. The two TI images were linearly registered onto the longest TI of the MP2RAGE image series with FLIRT in FSL. The dSIR processing incorporates a set of IR‐filters [17, 18] as follows: first, the so‐called Subtracted IR (SIR) T1‐filter, which involves subtracting TI = 600/1000 ms images from TI = 300/600 ms images (subtracted images obtained); second, the so‐called Added IR (AIR) filter by adding T1 = 300/600 ms and TI = 600/1000 ms images (added images obtained); and finally, the so‐called T1‐bipolar filter (BF) is applied by dividing the subtracted images by the added images to yield the dSIR images. It should be noted that TI = 600/1000 ms images were subtracted from TI = 300/600 ms images as opposed to the other way around [19] to obtain low dSIR signal intensity in tissue with short T1. Signals of species with varying T1 have different slopes between signal intensities at TI = 300/600 ms and TI = 600/1000 ms. Consequently, magnetisations of short T1 species have a steeper slope of vector between TI = 300/600 ms and TI = 600/1000 ms than that of long T1 species. The steeper slope which will be ‘amplified’ by the SIR filter. The slightly downward sloping vector of short and long T1 species at the null point reduces magnetisation when the AIR filter is applied. The BF filter produces the final amplification of magnetisation difference, when the SIR filtered image is divided by the AIR filtered one [19].

### 2.5 Segmentation of the Corpus Callosum (CC)

We segmented the CC for the ROI analyses using the JHU ICBM 1‐mm atlas [31] as previously described [8]. Briefly, the genu, midbody and splenium were segmented first from the atlas and then the midbody was manually segmented into three subregions, the anterior midbody, the posterior midbody and the isthmus [31] to match the reported axon diameter distribution as closely as possible [32]. The masks of the five subregions of the CC were registered to the native T1 space by registering the JHU‐ICBM 1‐mm FA map to the respective maps, and by applying the corresponding transformations to these CC masks with FLIRT and FNIRT in FSL [33]. These the so‐called large ROI masks project laterally between 19 and 26 mm from the midsagittal line. A second set of five CC masks by coverage to 4 mm on both sides of the midsagittal line were created, the so‐called midsagittal ROIs. For analyses of quantitative MRI measures the midsagittal ROI masks were subtracted from the large ROI masks to generate the so‐called lateral ROI masks. Anatomical match of the masks with the CC in T1 maps were visually verified. If needed the masks were corrected by 1–3 voxel layers to eliminate overlap with surrounding GM and/or CSF. T1, dSIR signal and diffusion microstructural measures were extracted from these masks using FSL [33].

## 3 Results

**FIGURE 1:**
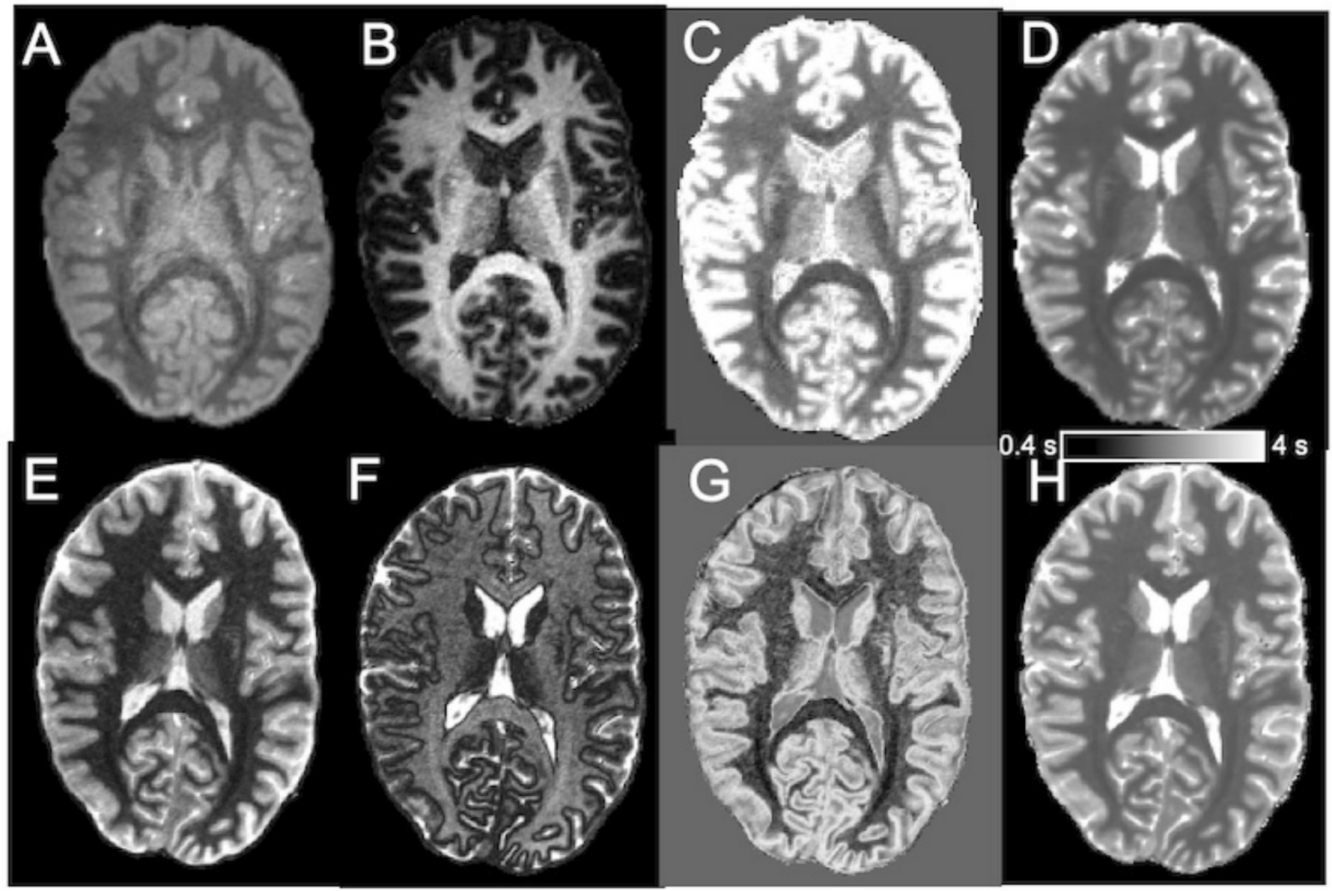
Typical axial MP2RAGE images used in dSIR processing, dSIR images and T1 maps at 3T (top row) and 7T (bottom row). Panel (A) shows typical axial MP2RAGE images acquired with TI = 300 ms and Panel (B) an image with TI = 600 ms at 3T. Panel (C) displays the dSIR image obtained by dividing the subtracted with the added image. Image in Panel (D) is a T1 map. Panel (E) shows a TI = 600 ms and in (F) a TI = 1000 ms image acquired at 7T. The respective dSIR processed image is shown in Panel (G) and panel (H) a T1 map at 7T. The horizontal rectangle (Panels D and H) is for T1 relaxation time reference from 0.4 to 4 s.

Typical TI = 300 ms and TI = 600 ms MP2RAGE images at 3T (Figure 1A,B) and TI = 600 ms and TI = 1000 ms images at 7T (Figure 1E,F) are shown that were used in the dSIR processing. dSIR images at 3T (Figure 1C) and 7T (Figure 1G) show bright GM and CSF relative to WM. It is conspicuous by eye that certain WM structures, such as in CC and inferior fronto‐occipital fasciculus, appear dark relative to the adjacent WM in the non‐windowed dSIR images (Figure 1C,G). Typical T1 maps are also displayed (Figure 1D,H). PSNR values in GM and WM in the TI and dSIR images are shown (Table 2). PNSR in WM in TI images were ~3‐fold and ~2‐fold greater than those in dSIR images at 3T and 7T, respectively. The low PSNR values in dSIR images are expected due to the noise bias resulting from combining magnitude images [19]. The PSNR in GM was ~2 times higher than that in WM at both fields. CNRs between GM and WM in each of the image type are given in Table 1S. CNR was comparable in dSIR images at both fields, but it was lower in AIR images at 3T than at 7T.

**TABLE 2:** PSNR in TI and dSIR images in human GM and WM at 3T and 7T.

Field	GM TI 1	GM TI 2	GM dSIR	WM TI 1	WM TI 2	WM dSIR
3T	20.7 ± 3.1	6.3 ± 1.2	12.7 ± 2.0	18.5 ± 2.4	17.8 ± 1.6	5.5 ± 2.6
7T	16.3 ± 1.8	8.3 ± 1.7	11.1 ± 2.4	12.9 ± 0.5	14.5 ± 1.5	5.4 ± 2.1

*Note:* PSNR was computed from the same GM and WM ROIs as used in the CNR analyses. TI 1 refers to TI = 300 ms at 3T and TI = 600 ms at 7T; TI 2 refers to TI = 600 ms at 3T and TI = 1000 ms at 7T. Values are mean ± SD from six volunteers at both fields.

**FIGURE 2:**
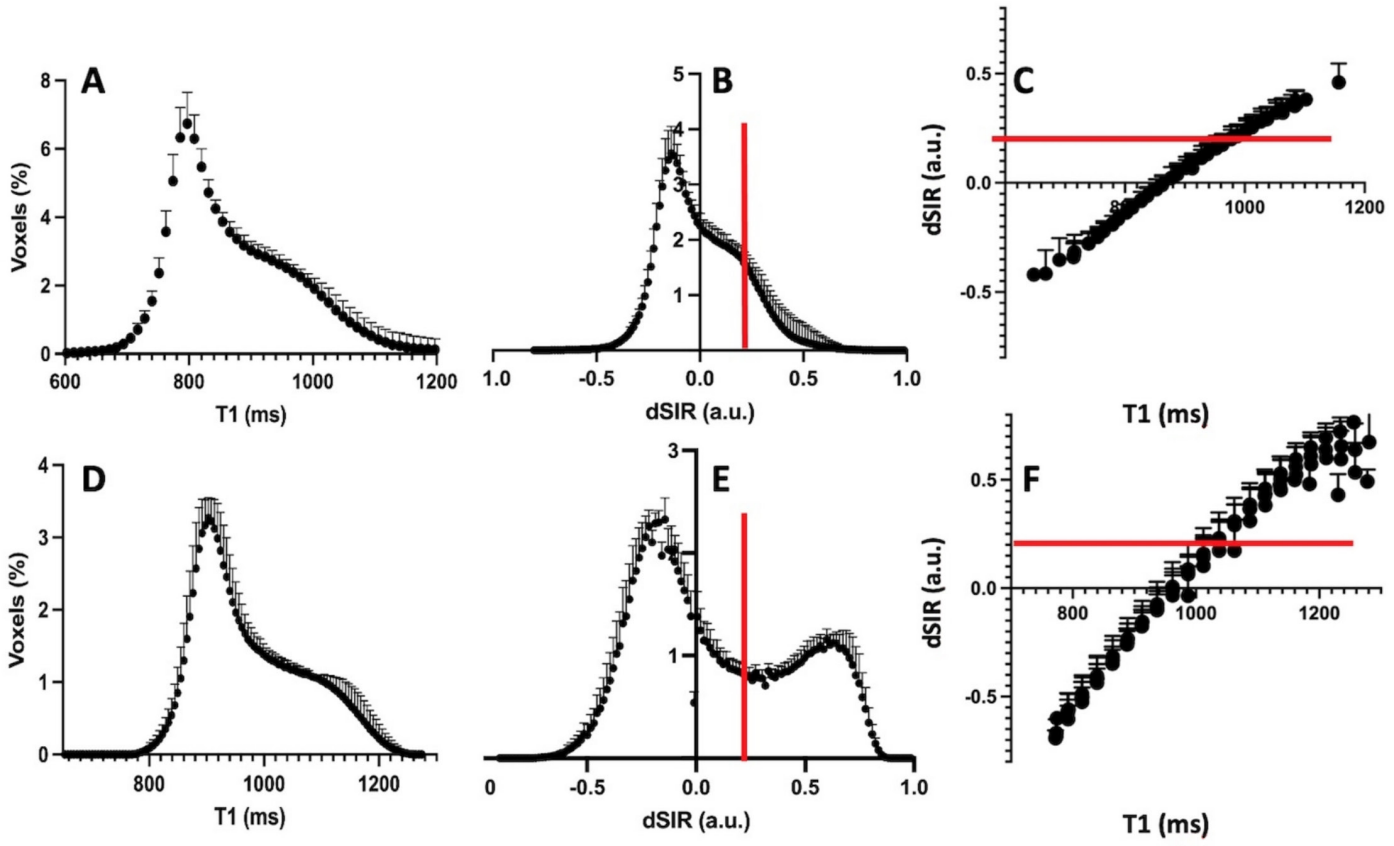
Voxel counts of T1 and dSIR signal intensities and dSIR signal as a function of T1 relaxation time in WM at 3T (top row) and 7T (bottom row). Voxel count distributions as a function of T1 relaxation time and dSIR signal intensities in anatomical WM at 3T (A and B) and at 7T (D and E). Panels (C) and (F) show dSIR signal intensity as a function of T1 (in ms) at 3T and 7T, respectively. The red vertical (B and E) and horizontal (C and F) lines show the cut‐off dSIR intensity used to remove the voxels of non‐linear relationship (C and F). FA was 0.501 ± 0.183 and 0.542 ± 0.214 in WM used for data at 3T and at 7T, respectively. Data are mean ± SD from six volunteers at both fields.

Voxel distributions according to T1 relaxation time (Figure 2A,D) and dSIR signal intensity (Figure 2B,E) in anatomical WM (FA was 0.501 ± 0.183 and 0.547 ± 0.214 at 3T and 7T, respectively) are shown. T1 ranged by about 400 ms in the anatomical WM (Figure 2A,D) both at 3T and 7T. The distributions of dSIR voxels at 3T showed a shoulder in the positive dSIR range (Figure 2B), whereas a bimodal distribution was evident at 7T (Figure 2E). At 3T 99.5% of dSIR voxels were within the intensity range of −0.6 to +0.6, while the corresponding percentage was 87.9% at 7T. Mean dSIR signal intensities in anatomical WM were 0.011 ± 0.191 and 0.096 ± 0.357 (n.s. *p* < 0.6159, Student's unpaired *t* test) at 3T and 7T, respectively, the largest number of voxels had dSIR intensity of around −0.1 at both fields. dSIR signal intensity versus T1 relaxation time plots (Figure 2C,F) show a non‐linear relationship, with non‐linearity becoming evident towards the positive end of dSIR values where T1 are long, which was likely due to the choice of TI images for dSIR processing [19]. However, the linearity was observed across negative dSIR values, though around a dSIR value of +0.2 the relationship started to bend. Linear regression analysis of dSIR in the range from −0.6 to +0.2 yielded formulas for 3T and 7T data as follows: *y* = 0.0021*x* − 1.8287 (*r* ^2^ = 0.9976) and *y* = 0.0034*x* − 3.2925 (*r* ^2^ = 0.9937). It should be noted that the slope of the 7T plot was 1.6 times greater than that of 3T, and that the difference in slopes may apply only to the current experimental conditions.

**FIGURE 3:**
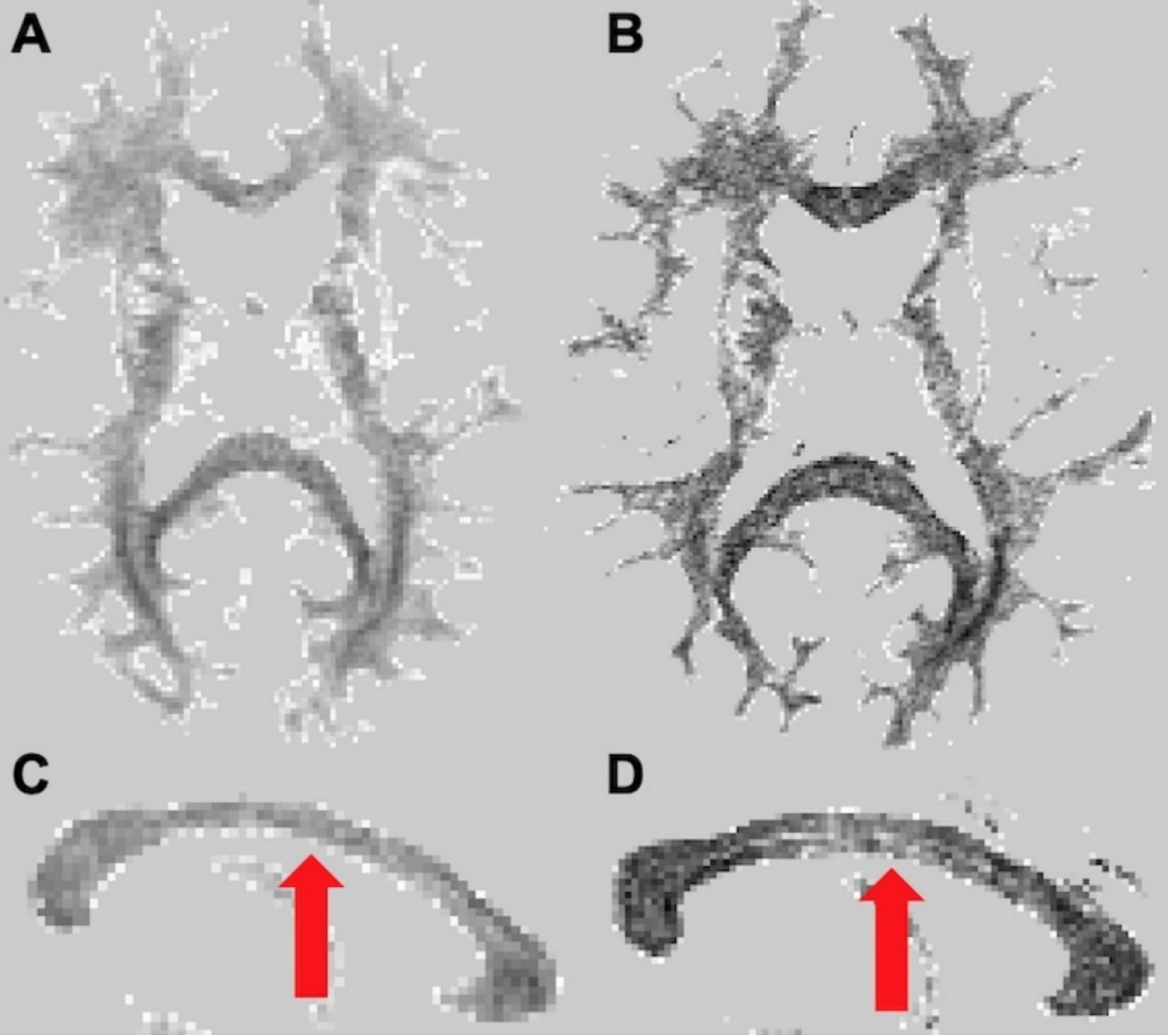
Typical dSIR images from a volunteer scanned at 3T and 7T. dSIR images were windowed from −0.8 to +0.2 to include the voxels from the dSIR voxels showing linear relationship with T1. Typical axial dSIR images at 3T (A) and at 7T (B) are shown. Panel (C) shows a midsagittal CC dSIR image at 3T and Panel (D) at 7T. The red arrows point to the somato‐motor subsections of CC where large and giant axons are present at high percentages.

Typical dSIR WM images thresholded from −0.8 to +0.2 are shown for 3T (Figure 3A,C) and 7T (Figure 3B,D). It is clearly visible that dSIR contrast within WM structures is stronger at 7T than at 3T, an effect, that depends on TI images used in the dSIR processing. For instance, in the genu and splenium of the CC and the fronto‐occipital fasciculus dSIR intensities were low relative to adjacent WM regions. Both in the midsagittal genu and splenium of the CC and fronto‐occipital fasciculus fibres are oriented close to perpendicular with respect to B0; thus, axon fibre orientation may be as a source of dSIR contrast.

**FIGURE 4:**
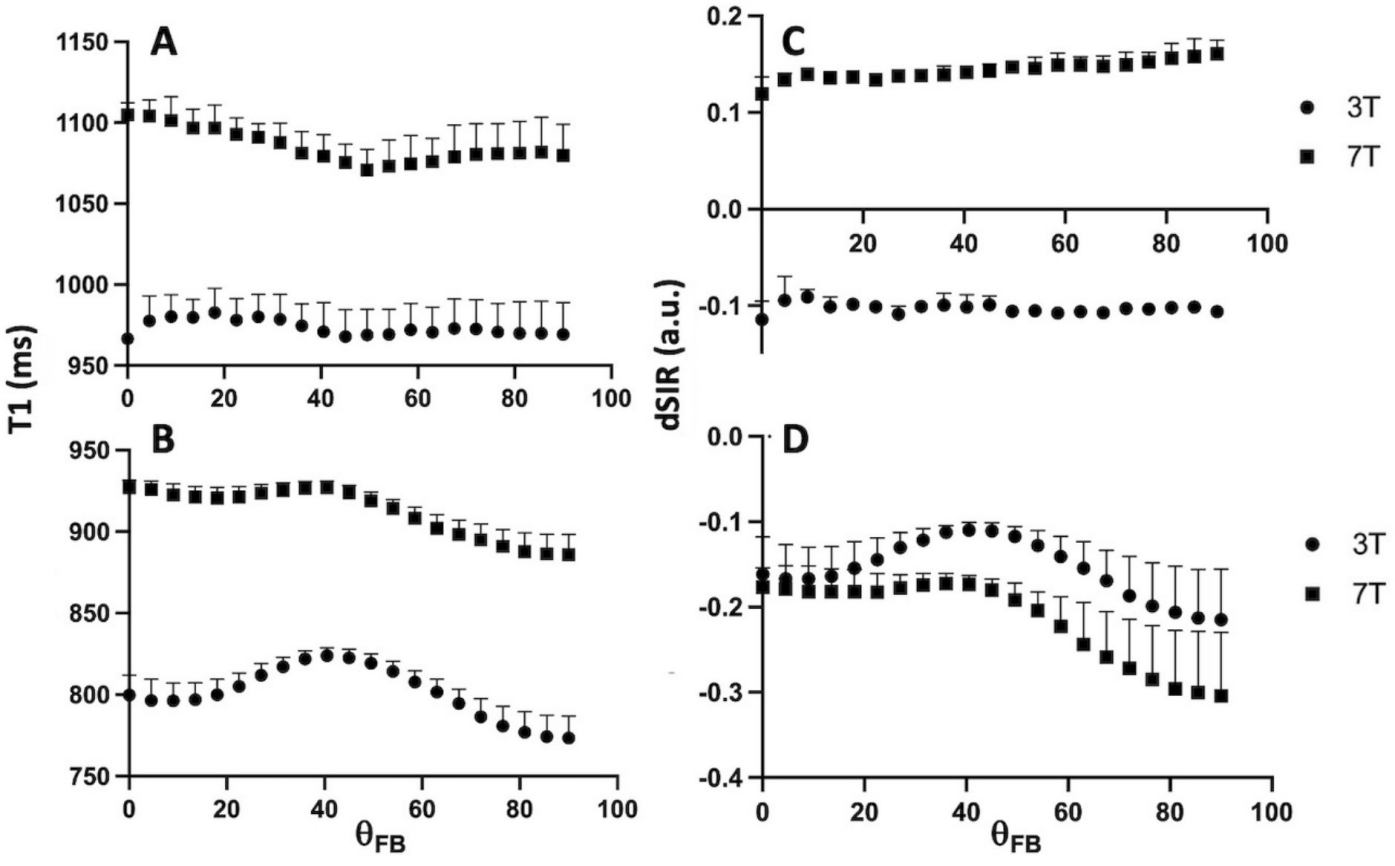
Angular plots of T1 and dSIR image intensity as a function of *θ*_FB_ in WM at 3T and 7T. Panel (A) shows T1 (in ms) as a function of *θ*_FB_ in low FA WM and Panel (B) in high FA WM at 3T (closed circles) and 7T (closed squares). Panel (C) displays dSIR image signal intensity as a function of *θ*_FB_ in WM with low FA WM and Panel (D) from WM in high FA at 3T and 7T, symbols as in Panel (A). Data in all panels are from six volunteers and are given as means ± SD.

The images above (Figure 3) prompted us to examine interrelationships between T1 relaxation time, dSIR image intensity and *θ*_FB_. The 1D plots between T1 and *θ*_FB_ (Figure 4A,B) and dSIR and *θ*_FB_ (Figure 4C,D) are shown. The plots for WM with low (FA = 0.250 ± 0.030, Figure 4A,C) revealed no consistent angular patterns between either T1 or dSIR and *θ*_FB_. In high FA WM (FA = 0.642 ± 0.100, Figure 4B) two angular features in T1 are present as follows: (a) shortening of T1 from fibre orientation of 0° to 90° and (b) a board hump with long T1 centred at 40°. Both these are consistent with the data in the previous reports [8, 13]. We note that the dSIR in high FA WM showed angular patterns that were tantalisingly similar to those seen in T1 at both fields (Figure 4D).

**FIGURE 5:**
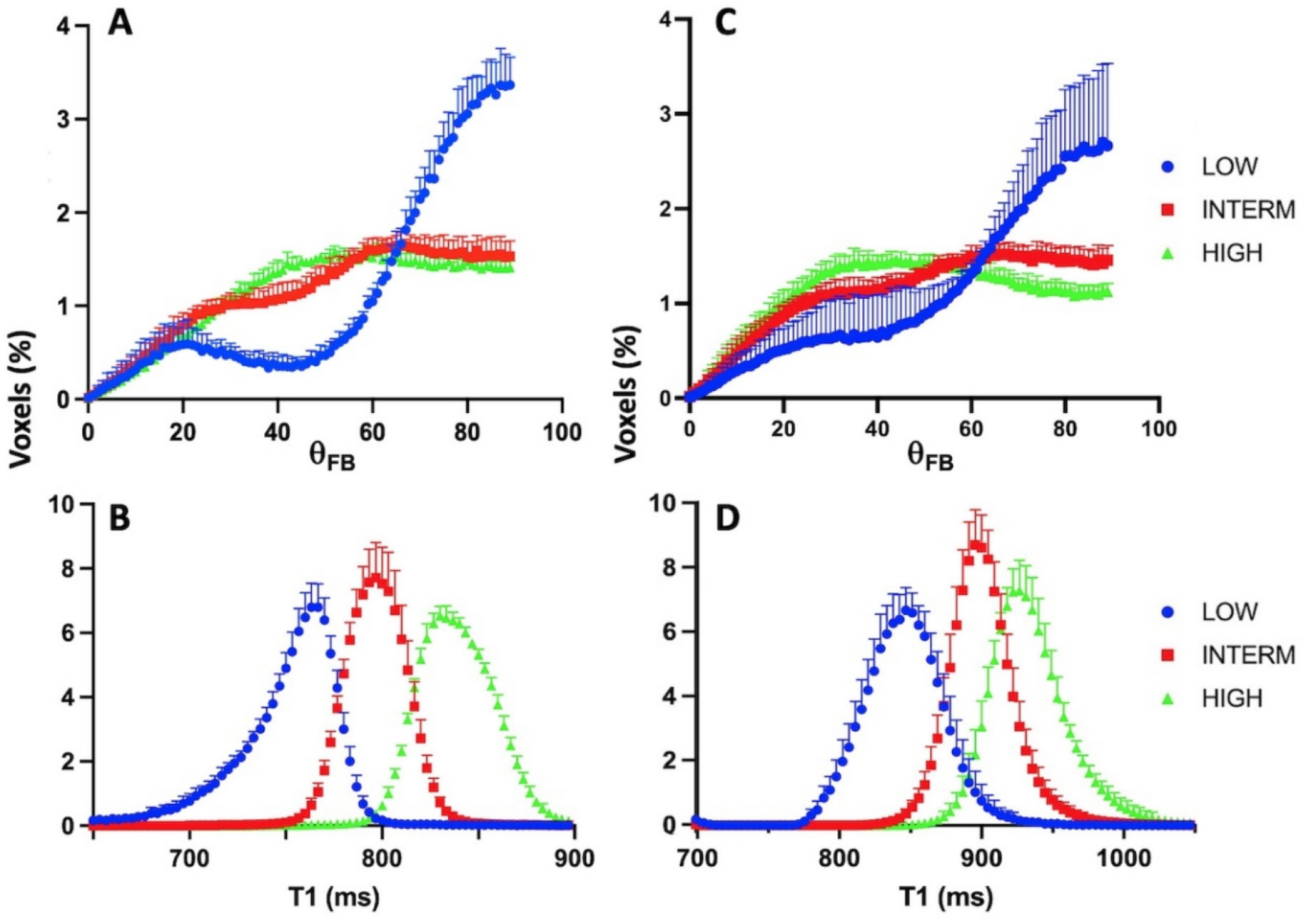
Distributions of low, intermediate and high dSIR signal voxels a functions of *θ*_FB_ and T1 in WM at 3T and 7T. In Panels (A) and (B), blue symbols mark voxels with dSIR signal intensities between (−0.9) and (−0.3) (LOW), red symbols show voxels with intensities between (−0.29) and (−0.15) (INTERM) and green symbols voxels with intensities between (−0.14) and (0.05) (HIGH). In Panels (C) and (D) blue symbols show voxels with dSIR signal intensities between (−0.9) and (−0.2) (LOW), red symbols show voxels with dSIR intensities between (−0.19) and (−0.1) (INTERM) and green marks voxels with dSIR intensities between (−0.09) and (0.02) (HIGH). Data are mean ± SD from six volunteers.

The data above indicate that the dSIR images may reveal contrast between WM tracts owing to their inherent fibre orientations. We examined how large differences in intratissue T1 relaxation times would be needed to generate dSIR contrast. To this end masks were created by binning dSIR images in low, intermediate and high dSIR signal intensity bins at 3T and at 7T. The cut‐offs for these bins were made as guided by the voxel distribution data (Figure 2B,E) so that approximately the same number of voxels were in each of the three bins. The voxel distributions in the three bins showed that the low dSIR signal bins had ~58% and ~49% of voxels with *θ*_FB_ between 70° and 90° at 3T and 7T, respectively, while the intermediate and high dSIR bins only had ~25%–30% of voxels in this *θ*_FB_ range at both fields (Figure 5A,C). Instead, in the intermediate and high signal bins *θ*_FB_ values were rather evenly distributed between 20° and 90° (Figure 5A,C). Histograms of voxels in the three bins show that T1 was shortest in the low dSIR signal and longest in the high dSIR signal WM, while the intermediate T1 was observed in intermediate dSIR signal WM at both fields (Figure 5B,D). These results reflect the interrelationships between the TI image timings used for dSIR processing and T1. Table 3 summarises numeric values for dSIR, T1, *θ*_FB_ and FA in the three bins.

**TABLE 3:** Quantitative MRI data from human white matter at 3T and 7T.

Parameter	Low dSIR (3T)	Intermediate dSIR (3T)	High dSIR (3T)	Low dSIR (7T)	Intermediate dSIR (7T)	High dSIR (7T)
dSIR (a.u.)	−0.267 ± 0.070*	−0.146 ± 0.028	−0.053 ± 0.028	−0.386 ± 0.074*	−0.220 ± 0.042	−0.082 ± 0.041
T1 (ms)	745.7 ± 43.5*	798.1 ± 25.8	842.2 ± 27.7	868.4 ± 78.8*	904.8 ± 62.8	940.9 ± 94.2
*θ*_FB_ (degree)	66.3 ± 21.4*	55.2 ± 20.9	54.8 ± 21.0	65.4 ± 20.3*	53.8 ± 22.4	49.9 ± 22.2
FA	0.582 ± 0.166*	0.512 ± 0.132	0.473 ± 0.130	0.619 ± 0.122*	0.572 ± 0.106	0.551 ± 0.100

*Note:* Image intensities in dSIR images of human WM were binned into three bins as follows: low dSIR from (−0.9) to (−0.2), intermediate from (−0.19) to (−0.1) and high from (−0.09) to (0.02) at 3T and low dSIR from (−0.9) to (−0.3), intermediate from (−0.29) to (−0.15) and high from (−0.14) to (0.05) at 7T. The masks from each of these bins were used to extract T1, fibre‐to‐field‐angle and FA values for the bin ranges at both fields.

* *p* < 0.01 low dSIR bin versus intermediate and high dSIR bins, paired Student's *t* test. Values are means ± SD from six volunteers.

**FIGURE 6:**
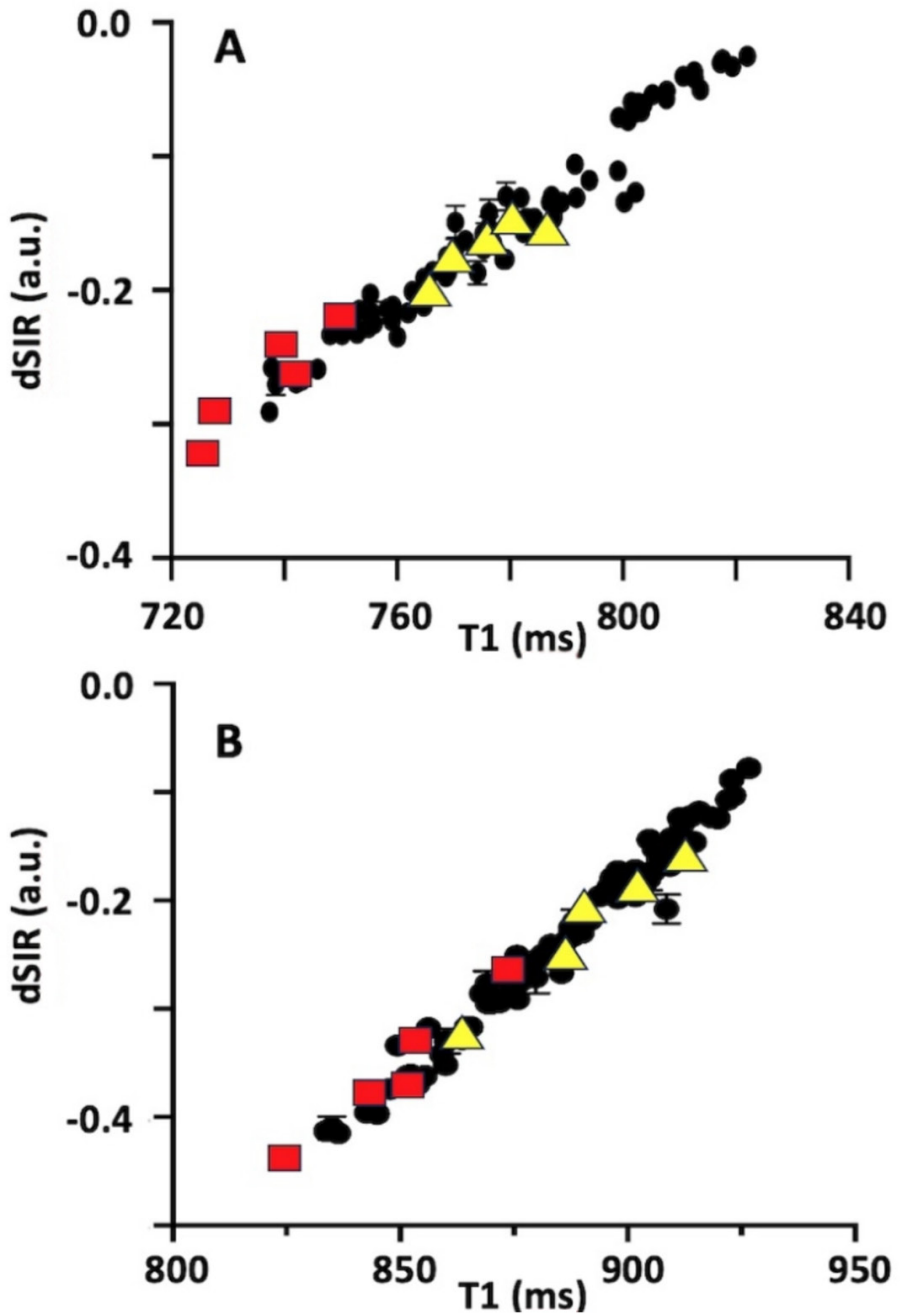
dSIR image intensity as a function of T1 in WM at 3T and 7T. Panel (A) show dSIR image signal intensities as a function of T1 analysed in the midsagittal and lateral subsections of the CC, in two sets of WM ROIs where *θ*_FB_ ranged from 0° to 20° and from 35° to 50°. Linear regression of data in Panels (A) and (B) gave formulas as follows: *y* = 0.0031*x* − 2.5325 (*r* ^2^ = 0.9534) and *y* = 0.0036*x* − 3.4526 (*r* ^2^ = 0.9772). In Panels (A) and (B) data from midsagittal ROIs of the genu are shown by red squares and from midsagittal somato‐motor ROIs by yellow triangles. Data in all panels are from six volunteers.

To further scrutinise the interrelationships between dSIR signal, T1 and *θ*_FB_, we examined these MRI variables in the midsagittal and lateral subsections of the CC where *θ*_FB_ varies over a short distance due to the inherent orientations of fibres within the same tracts [8]. In the midsagittal CC fibres are close to perpendicular to B0 (*θ*_FB_ > 75°) whereas in lateral ROIs fibres have up to 20° smaller *θ*_FB_ than those midsagittally [8]. dSIR, T1 and *θ*_FB_ were quantified in midsagittal and lateral subsections of the CC as well as in WM ROIs with *θ*_FB_ ranging from 0° to 20° and 35° to 50° orientations. Plots of dSIR signal intensities as a function of T1 showed linear relationships at both fields with *r* ^2^s of 0.9534 and 0.9772 (Figure 6A,B). At 3T the dSIR versus T1 slope was 0.00306 and at 7T the slope was 1.2 times greater at 0.00368. Figure 3C,D showed brighter dSIR signal in midsagittal somato‐motor subsections of the CC than in the genu. In the dSIR versus T1 plots data points from ROIs of midsagittal genu and somato‐motor are marked with red squares and yellow triangles (Figure 6A,B), respectively. Greater proportions of large and giant axons are present in the latter subsection of the CC than in the former [32] and because T1 in the latter is longer than in the former [5, 34]. The dSIR data from the two midsagittal subsections of the CC clustered so that in the somato‐sensory subsection with longer T1 dSIR values were greater than those in the genu. dSIR values in genu and somato‐motor subsections were −0.243 ± 0.145 and −0.160 ± 0.136 (*p* < 0.01, paired Student's *t* test) at 3T for a T1 difference (ΔT1) of 29.3 ± 9.0 ms. The respective values at 7T were −0.343 ± 0.167 and −0.211 ± 0.167 (*p* < 0.01, paired Student's *t* test) for ΔT1 of 41.7 ± 13.3 ms.

## 4 Discussion

The data demonstrate that the dSIR approach, where a pair of closely timed TI images is arithmetically processed, provides contrast without intensity windowing where a small variation in intratissue T1 in WM becomes visible. We observed linear relationships between dSIR signal intensity and WM T1 over a range of approximately 200 ms both at 3T and 7T. The dSIR signal in WM showed angular dependencies that closely matched those of T1 relaxation time [8, 12]. These observations strongly argue that the dSIR images closely mimic T1 in agreement with the previous studies [18, 19]. dSIR images provide intratissue contrast due to small T1 variation and thereby bear the potential to visualise WM microstructural features to complement other MRI techniques for microstructure imaging in vivo. The dSIR approach may be able to visualise the effects of fibre orientation [6, 11, 12] as well as fibre configuration and structural anisotropy [9], and axon diameter [5] in a convenient manner with IR MRI sequences.

Combining images acquired at high resolution in a time efficient manner became commonplace following introduction of the MP2RAGE method [2]. The T1‐weighted images by MP2RAGE, obtained by combining a TI image pair typically 1500 to 2000 ms apart, one with T1‐weighting and another with proton density weighting, provide excellent GM/WM contrast, in addition to that MP2RAGE images are immune to bias field, T2* and proton density artefacts. The GM/WM contrast in MP2RAGE images results from a typical ΔT1 of ~400 ms at 3T and ~500 ms at 7T between the tissue types. The dSIR approach coined by Bydder and coworkers [19, 30] utilises images from a TI image pair typically 150 ms apart at 3T [19], with TIs chosen from both sides of the null point of the target T1s. The essence of the dSIR contrast has been recently understood via validation using a quantitative T1 phantom at 3T [19]. The dynamic range in dSIR images varies between −1 and +1 thereby covering a wide range of T1s in a non‐linear fashion [17]. Non‐linearity is concentrated to both the short and long ends of T1s covered. Our approach basically follows the dSIR protocol with some exceptions both in TI images acquisitions and image processing itself. First, we used 3D MRI instead of a 2D acquisition, and in our images a gradient echo readout was used instead of FSE [17, 19]. Use of different pulse sequences results in non‐equal absolute T1 values [35], a fact that must be taken into considerations when choosing TI times for dSIR. Second, in the image processing we subtracted long TI images from short TI images to obtain low dSIR signal intensity in tissue with short T1, our ΔTI was wider (300/400 ms) than that used by Bydder et al. (150 ms) at 3T [19], and we used the NIFTI image format rather than DICOM [19]. The use of wide ΔTI in the current study inevitably weakened dSIR‐contrast‐to‐short‐TI‐contrast‐ratio [17]. Therefore, the TI times used here were not as optimal for dSIR image quality as those processed by Bydder and coworkers [19]. Because ΔTI was wider at 7T than at 3T, the full benefit of ultrahigh field for dSIR contrast was not fully achieved. Nevertheless, the 7T dSIR images obtained gave excellent intratissue contrast in WM outperforming that obtained at 3T.

The dSIR approach to visualise subtle effects by imminent pathology in the human brain [17] has been estimated to result from a minimal ΔT1 of ~100 ms between normal and pathological WM at 3T [19]. The current data indicate that even smaller intratissue ΔT1 are sufficient to produce dSIR intratissue contrast. ΔT1 of order of ~50 ms results from differing fibre orientations, for instance between midsagittal and lateral fibres in CC tracts and are visualised by dSIR images (such as in Figure 3A,B). Similarly, ΔT1s of ~50 ms were measured between WM bins containing large percentage of fibres with high *θ*_FB_ relative to the WM where fibres are evenly oriented, and these two types of WM were separated by dSIR (e.g., Figure 5 and Table 3). ΔT1s on the order of ~50 ms exist also between WM regions consisting of single and complex fibre configurations, such as those in the corona radiata and genu of the CC [9]. We measured T1 that was longer in the somato‐motor CC with greater percentage of large and giant axons [32] than in the genu by ~30 and ~40 ms at 3T and 7T (Figure 6A,B) and that dSIR signal was greater in the former than in the latter subsection of the CC. These observations argue that ΔT1s as small as 30–50 ms are sufficient to yield intratissue dSIR signal differences in WM.

A generic constraint of combining two magnitude images includes the introduction of noise bias [36] as quantitatively evaluated in connection to dSIR processing [19]. We observed inferior PSNR in dSIR images relative to those in TI images. Nevertheless, it is worth noting that image quality obtained was such that the dSIR signal ‘in physical units’ could be used as surrogate to small T1 differences in WM resulting from microstructural features. A further point to be noted is that PSNR in both tissue types were similar at both fields despite the 2.6‐fold smaller voxel volume at 7T than at 3T. Thus, the gain in sensitivity afforded by 7T can also be exploited in high spatial resolution also for dSIR images. In addition, there are limitations related to the current study, such as that the MP2RAGE parameters used were primarily targeted to acquire IR images to compute T1 maps, not specifically for the optimised separation of T1s used by the dSIR approach [19]. The ΔTIs we used were wider than the optimal ones leading to non‐optimised dSIR images [30]. Further, we directly measured the effects of FA, fibre orientation and large and giant size axons on the dSIR signal as the WM microstructural features. It is well known that myelination influences T1 [37], yet we do not have direct estimates for myelin content in the current images. Finally, there are well appreciated issues in noise addition due to arithmetic processing of magnitude images which inevitably will place limits to the dSIR image quality as pointed out recently [19].

To conclude, the current study demonstrates that dSIR processing of a pair of closely separated inversion time images produces images where signal intensity quantitatively mirrors T1. The influence of microstructure on non‐windowed dSIR contrast is sufficient to visualise effects of microstructural features in WM.

## Supporting information


**Table S1** CNR in TI and dSIR images at 3 T and 7 T

## Data Availability

The data that support the findings of this study are available from the corresponding author upon reasonable request.
