# Supplementary material for: A Subtracted‐Added‐Divided Inversion Recovery (dSIR) Approach to Visualise the Effects of Microstructure on T1 Contrast in Human White Matter
Source: NMR Biomed. 2025 May 29;38(7):e70070. doi: 10.1002/nbm.70070 (PMC12120812; doi:10.1002/nbm.70070)
Supplement: Supplementary file 1 — Table S1 CNR in TI and dSIR images at 3 T and 7 T [file NBM-38-e70070-s001.docx]

**SUPPLEMENTARY MATERIAL NBM-25-0158**

**A SUBTRACTED-ADDED-DIVIDED INVERSION RECOVERY (dSIR) APPROACH TO VISUALISE THE EFFECTS OF MICROSTRUCTURE ON T1 CONTRAST IN HUMAN WHITE MATTER**

Risto A. Kauppinen^1^, Jeromy Thotland^2^, Pramod K. Pisharady^2^, Christophe Lenglet^2^ and Michael Garwood^2^

^1^Department of Electric, Electronic and Mechanical Engineering, University of Bristol, Bristol, UK; ^2^Center for Magnetic Resonance Research, University of Minnesota, Minneapolis, MN, USA.

**Table 1S.** CNR in TI and dSIR images at 3T and 7T

| **FIELD** | **TI 1** | **TI 2** | **dSIR** | **SIR** | **AIR** |
| --- | --- | --- | --- | --- | --- |
| **3T** | 7.0±2.1 | 7.3±1.1 | 7.2±1.3 | 8.4±1.3 | 2.4±0.6 |
| **7T** | 7.9±2.1 | 0.94±0.83 | 5.2±2.5 | 6.5±1.6 | 6.1±3.8* |

Signals intensities and SD of signals in two brain tissue types were measured in parietal ROIs of 3x3x3 voxels at both fields. TI 1 stands for TI=300ms at 3T and TI=600ms at 7T, TI 2 for TI=600ms at 3T and TI=1000ms at 7T. ‘SIR’ and ‘AIR’ refer to subtracted and added images, respectively, in the dSIR processing. Values are mean ± SD from 6 volunteers at both fields. * indicates p<0.05, Student’s unpaired t-test, 3T value vs 7T value.
